# Antimicrobial Peptides from Fruits and Their Potential Use as Biotechnological Tools—A Review and Outlook

**DOI:** 10.3389/fmicb.2016.02136

**Published:** 2017-01-10

**Authors:** Beatriz T. Meneguetti, Leandro dos Santos Machado, Karen G. N. Oshiro, Micaella L. Nogueira, Cristiano M. E. Carvalho, Octávio L. Franco

**Affiliations:** ^1^S-Inova Biotech, Programa de Pós-Graduação em Biotecnologia, Universidade Católica Dom BoscoCampo Grande, Brazil; ^2^Graduação em Ciências Biológicas, Universidade Católica Dom BoscoCampo Grande, Brazil; ^3^Centro de Análises Proteômicas e Bioquímicas, Pós-Graduação em Ciências Genômicas e Biotecnologia, Universidade Católica de BrasíliaBrasília, Brazil

**Keywords:** antimicrobial peptides, biotechnological potential, fruits, infections, microorganisms

## Abstract

Bacterial resistance is a major threat to plant crops, animals and human health, and over the years this situation has increasingly spread worldwide. Due to their many bioactive compounds, plants are promising sources of antimicrobial compounds that can potentially be used in the treatment of infections caused by microorganisms. As well as stem, flowers and leaves, fruits have an efficient defense mechanism against pests and pathogens, besides presenting nutritional and functional properties due to their multifunctional molecules. Among such compounds, the antimicrobial peptides (AMPs) feature different antimicrobials that are capable of disrupting the microbial membrane and of acting in binding to intra-cytoplasmic targets of microorganisms. They are therefore capable of controlling or halting the growth of microorganisms. In summary, this review describes the major classes of AMPs found in fruits, their possible use as biotechnological tools and prospects for the pharmaceutical industry and agribusiness.

## Introduction

Intensive and prolonged antimicrobial therapy, and the over-prescription and indiscriminate use of these drugs in veterinary medicine, have increased the resistance of microorganisms to conventional antimicrobials by selection pressure, so it is urgently necessary to search for new alternatives to these drugs. For bacteria to remain in the human organism, they have developed several defense mechanisms against antibiotics (Podschun and Ullmann, 1998). The selection pressure on susceptible microbes, the long duration and over-prescription of antimicrobial therapy and the use of various antimicrobial agents in animals raised commercially for food has made it necessary to search for new alternatives to conventional antibiotics. Thus, the types of antibiotics already commercialized and new combinations that may be available among them (Gordon et al., 2010) should be analyzed, so that they may present higher antibacterial activity against infections and biofilms (Corvec et al., 2013).

Like many living organisms, plants are constantly targets of insects, fungi and bacteria. These ongoing challenges can be responsible for the development of an efficient defense system through the synthesis of secondary metabolites such as phenols, oxygen-substituted derivatives, terpenoids, quinines, tannins, and antimicrobial peptides (AMPs) (Abreu et al., 2013). AMPs can be isolated from a wide variety of plants or parts of plants (leaves, roots, seeds, flowers, and fruits); they are considered an important part of the innate immune system and act as a complex signaling process (Maróti et al., 2011). These compounds can be classified as promiscuous proteins due to their ability to interact with several targets in Gram-positive and -negative bacteria, protozoa, yeast, fungi, and viruses (Franco, 2011). Moreover, they can also be associated in inflammatory response, infectious diseases, immunosuppressive, and tumoral diseases (Jenssen et al., 2006; Palffy et al., 2009; Rotem and Mor, 2009; Brogden and Brogden, 2011). In addition to antibiotics, environmental conditions and evolutionary pressure have made microorganisms capable of changing cellular structures or producing substances in order to contain antimicrobial peptide action (Brogden, 2005). Among the main resistance mechanisms are membrane structure modifications (Tran et al., 2006); peptide capture (Banemann et al., 1998; Friedrich et al., 1999); and capsule formation (Campos et al., 2004; Llobet et al., 2008; Jones et al., 2009; Keo et al., 2011). In this last mechanism, the capsular polysaccharide may act as a shield, blocking interaction between the cAMPs and the target cell. Capsule formation can also be performed by a dissociated polymer of *P. aeruginosa*, denominated an alginate, which changes the AMP's conformation, blocking its interaction with the target cell (Chan et al., 2004). Or it can be carried out by an LPS activation enzyme, which is the main component of Gram-negative cells, forming a regulatory system of PhoPQ-PmrAB and PhoP-PhoQ, so that the presence of environmental factors, such as cAMPs, can be detected in host tissues (Muhle and Tam, 2001; McPhee, 2003; Moskowitz et al., 2004; McPhee et al., 2006; Strandberg et al., 2012).

Storage organs and reproductive tissues of fruits are responsible for the production and accumulation of related peptides in the first line of defense (Tajkarimi et al., 2010; Hayek et al., 2013). They may be a source of bioactive compounds that have safer side effects when used in the prevention and control of plant pathogens and pests (Broekaert et al., 1997; Guani-Guerra et al., 2009) and of human diseases (Memarpoor-Yazdi et al., 2013). These effects can be attributed to the fact that AMPs have a mechanism of action that is significantly different from conventional antibiotics. They are natural molecules and are essential for the innate immune system (Fox, 2013). The AMPs' promising activity is due to their great applicability as antimicrobials, and they could probably be used in synergism with other drugs, creating immunomodulatory side-activities or neutralizing toxic compounds including LPS, presenting a lower minimum inhibitory concentration (MIC) than conventional antibiotics (Zasloff, 2002; Naghmouchi et al., 2012). The safer effect of AMPs may be related to their ability to act on multi-resistant microorganisms, rapidly causing the death of these pathogens and being capable of dealing with large bacterial targets (Brogden, 2005). Due to the high resistance of some infections to traditional antibiotics, AMPs with their broad spectrum antibacterial activity are highly promising in the treatment of these infections. Among them are included colistin and polymixin B, which are currently used widely in clinical practice (Falagas and Kasiakou, 2005; Zavascki et al., 2007; Landman et al., 2008). Additionally, the probability of resistance and mutagenicity being induced by AMPs may be lower in natural environments (Perron et al., 2006; Dobson et al., 2014). AMPs generated in fruits may therefore be a new target for bioprospecting new molecules with multiple targets in microorganisms and resistance frames that are relatively small compared to conventional antimicrobials (Jenssen et al., 2006). In this context, this review aims to describe the different classes of AMPs isolated from the pulp and seeds of fruit and to provide an outlook on their biotechnological potential.

## Fruit antimicrobial peptides

AMPs are small (<10 kDa) and normally have cationic and amphipathic molecules (one surface being highly positive and the other hydrophobic and responsible for facilitating the bond between the peptide and the target membrane) (Brogden, 2005; Kang et al., 2012; Kościuczuk et al., 2012; Seo et al., 2012). These molecules can act in the cytoplasmic membrane through rupture, disintegration and formation of pores (Brogden, 2005; Nguyen et al., 2011; Li et al., 2012). In addition to the mechanisms of action related to the plasmatic membrane, cationic peptides can also act by inhibiting protein transport or enzymes (Broekaert et al., 1997; Carvalho and Gomes, 2001; Lay and Anderson, 2005), interacting with DNA, RNA, inhibiting ion channels (Kushmerick et al., 1998; Spelbrink et al., 2004), acting in the regulation of steroid hormone (Huang et al., 2013) and development of potential redox (Takayama et al., 2001; Stotz et al., 2009; Amien et al., 2010), and inhibiting peptidoglycan synthesis (Yeaman and Yount, 2003).

AMPs have been grouped into various families by the APD3 database (http://aps.unmc.edu/AP/main.php), so they can be divided, based on 3D structures, into (Wang et al., 2016): (I) linear -helical peptides; (II) cyclic peptides with β-sheet structures with two or more disulfide bonds; (III) a combination of α-helices and β-sheets stabilized by disulfide bonds; (IV) peptides with -hairpin or looped arrangement containing disulfide bonds; (V) linear peptides with an unusual predisposition for the particular repetition of some amino acid residues, including proline, glycine, tryptophan, or histidine; and (VI) short peptides with coil structures or with no defined secondary structures. Additionally to this classification, several classes of plant AMPs can also be divided by properties, such as covalent bonding patterns, hydrophobicity, net charge, or molecular targets (Wang, 2015). Among several different plant AMP classes, in fruits it is possible to detect (I) defensins (Meyer et al., 1996; Guzmán-Rodríguez et al., 2013; Seo et al., 2014), (II) lipid transfer proteins (LTPs) (Zottich et al., 2011), (III) glycine rich protein (Pelegrini et al., 2008), (IV) 2S albumin (Pelegrini et al., 2006; Ribeiro et al., 2011), (V) snakin (Daneshmand et al., 2013), (VI) napin (Da Silva Dantas et al., 2014), and (VII) and other fruit AMPs (Table 1).

**Table 1:** **Classes of AMPs, source and name of peptides and their rate (MICs) and percentage of inhibition**.

**Class**	**Source**	**Peptide**	**Antimicrobial Activity**	**MICs (μg.mL^−1^)**	**Concentration (μg.mL^−1^)/% m of inhibition**	**Reference**
Defensin	Avocado (*P. americana*)	*Pa*Def	*E. coli*	–	10^2^/78.08	Guzmán-Rodríguez et al., 2013
			*S. aureus*	–	10^2^/67.43	
	Fruit peppers (*C. annuum* var. *Yolo Wonder*)	J1	*C. gloeosporioide*	–	10^−4^/50%	Seo et al., 2014
			*C.musae*	–	1.5 × 10^2^/100%	
			*F. oxysporum*	–	1.5 × 10^2^/100%	
			*C. albicans*	–	1.5 × 10^2^/100%	Diz et al., 2006
			*S. cerevisiae*	–	1.5 × 10^2^/100%	
			*S. pombe*	–	1.5 × 10^2^/100%	
						
Lipid Transfer Proteins (LTPs)	Chili pepper *(C. annuum L.)*	*Ca*-LTP1	*C.tropicalis*	–	4 × 10^2^/70%	Diz et al., 2011
			*F. oxysporum*	–	10^2^	
			*C. lindemunthianum*	–	10^2^	
			*S. cerevisiae*	–	8	
			*P. membranifaciens*	-	8	
			*C. tropicalis*	–	8	
			*C. albicans*	–	8	Cruz et al., 2010
2S Albumin	Passion fruit (*P. alata* Curtis)	*Pa*-AFP1	*C. gloeosporioides*	–	10^2^/70%	Ribeiro et al., 2011
	Passion fruit (*P. edulis)*	*Pe*-AFP1	*T. harzianum*	–	80%	Pelegrini et al., 2006
			*A. fumigatus*	–	60%	
			*F. oxysporum*	–	70%	
	Passion fruit (*P. edulis* f. flavicarpa)	*Pf*2	*F.oxysporum*	–	6 × 10/24%	Agizzio et al., 2003
			*C.musae*	–	6 × 10/32%	
			*S. cerevisiae*	–	6 × 10/32%	
			*C. lindemuthianum*	–	6 × 10/78%	
Glycine-rich protein	Guava seeds (*P. guajava*)	*Pg*-AMP1	*Klebsiella* sp	7.2 × 10	4 × 10/90%	Pelegrini et al., 2008
			*Proteus*	–	4 × 10/30%	
			*E. coli*	3.2 × 10	–	
Snakin	Jujuba fruits (*Z. jujuba*)	Snakin-*Z*	*A. niger*	9.3	–	Daneshmand et al., 2013
			*C. albicans*	8.23	–	
			*P. azadirachtae*	7.65	–	
			*P. ultimum*	8.36	–	
			*S. aureus*	2.88 × 10	–	
			*E. coli*	1.36 × 10	–	
			*K. pneumoniae*	1.41 × 10	–	
			*B. subtilis*	2.42 × 10	–	
Napin	Jambo fruit (*E. malaccensis*)	*Em*2-F18	*S. aureus*	1.5 × 10^−1^	98%	Da Silva Dantas et al., 2014
			*S. enterica Enteritidis*	1.5 × 10^−1^	40%	
	Coconut water *Trapa natans*	*Tn*-AFP1	*F. oxysporum*	–	–	Wang and Ng, 2005
			*M. arachidicola*	–	–	
	Green coconut (*C. nucifera* L)	*Cn*-AMP1	*P. piricola*	–	–	
			*C. tropicalis*	3.2 × 10	–	
		*Cn*-AMP2	*E. coli*	8.2 × 10	–	Mandal et al., 2011
			*P. aeruginosa*	7.9 × 10	–	
			*S. aureus*	8.0 × 10	–	
			*B. subtilis*	7.6 × 10	–	
Unclassified AMPs from Fruit			*E. coli*	1.70 × 10^2^	–	
		*Cn*-AMP3	*P. aeruginosa*	1.69 × 10^2^	–	
			*S. aureus*	1.70 × 10^2^	–	
			*B. subtilis*	1.50 × 10^2^	–	
			*E. coli*	3.02 × 10^2^	–	Mandal et al., 2009
			*P. aeruginosa*	2.59 × 10^2^	–	
			*S. aureus*	2.74 × 10^2^	–	
			*B. subtilis*	2.57 × 10^2^	–	

### Defensins

Defensins are apparently ubiquitous throughout the plant kingdom. They can mainly be included in the families of Brassicaceae, Fabaceae, and Solanaceae. Defensins are small (12–45 amino acids) with approximately 5 kDa, highly basic and include 8–10 cysteines involved in disulfide bridges that have the function of stabilizing these molecules (Kobayashi et al., 1991; Zhu et al., 2005). In plant defesins, two types of precursors were identified and described in a class where the dominant group is composed of the N-terminal signal peptide (Finkina et al., 2008), and the lesser group is composed of an extra C-terminal acidic pro-domain associated with a vacuolar sorting mechanism (Terras et al., 1995; Lay et al., 2003; Lay and Anderson, 2005). Differently from mammalian defensins, plant defensins have a well-conserved three-dimensional structure, being stabilized for specific Cys composition, which involves a structure stabilized by four disulfide bonds (Cys1–Cys8, Cys2–Cys5, Cys3–Cys6 and Cys4–Cys7) (Lay and Anderson, 2005). There are two Gly residues at positions 13 and 34, in addition to the conserved Cys residues, a Glu residue at position 29 and a conserved aromatic residue at position 11 (Broekaert et al., 1995; Meyer et al., 1996; Artlip and Wisniewski, 2001). Studies of their three-dimensional structure have shown that it comprises a triple-stranded β-sheet with an α-helix in parallel (Bruix et al., 1993, 1995; Bloch et al., 1998; Fant et al., 1998, 1999; Almeida et al., 2000). They can be expressed during storage and reproduction, being related to antibacterial and antifungal activities (Broekaert et al., 1997; Wijaya et al., 2000; Stotz et al., 2009), environmental stress response (Maitra and Cushman, 1998), as well as signaling molecules, including methyl jasmonate, ethylene, and salicylic acid (Hanks et al., 2005), and regulating the innate immune system (de Beer and Vivier, 2011).

The defensins can be expressed during the period of storage and reproduction. One example is defensin J1 from bell pepper (*Capsicum annuum* var. yolo) (Meyer et al., 1996). Northern and Western blot analysis revealed that J1 could be accumulated during maturing phases. Likely to play a role in host defense, the expression from J1 during these phases seems to protect the fruits against pathogens, increasing fruit integrity and ensuring seed maturation. Antifungal activity of J1 was assessed by disk diffusion assay against *Fusarium oxysporum* and *Botrytis cinerea* (Meyer et al., 1996). Seo et al. (2014) proved that the J1-1 peptide is overexpressed during its development in fruits, mainly. Furthermore, fruits infected with *Colletotrichum gloeosporioides* had more production than ininfected fruits. In the same study, the J1-1 peptide was able to inhibit 50% of the growth of the *C. gloeosporioide*s at 1 mg.mL^−1^.

Similary to other defensins, the *Ppdfn1* gene identified in peach (*Prunus persica*) showed a strong antifungal activity (Nanni et al., 2013). *Ppdfn1* can act against *B. cinerea, Monilinia laxa*, and *Penicillium expansum*, with IC_50_ values of 15.1, 9.9, and 1.1 μg.mL^−1^, respectively. This peptide is localized on the external cell surface where it is capable of membrane destabilization and permeabilization. Analysis of transcript levels and their accumulation were showed in several times throughout development indicated that PpDfn1 is seasonally expressed in early fruit development. Though a recombinant version, *rDFN1*, expressed in the yeast *Pichia pastoris*, inhibited the germination of *P. expansum* and *B. cinerea*, it does not have antimicrobial activity against the Gram-negative bacterium *Erwinia amylovora* (Wisniewski et al., 2003).

Another defensin, named *Pa*Def, founded in avocado fruit (*Persea americana* var. drymifolia.), showed antibacterial activity against Gram-positive and negative bacteria. *Pa*Def should inhibit 67.43 and 78.08% of the viability of *Escherichia coli* and *Staphylococcus aureus*, respectively, in concentrations for 10 at 100 μg.mL^−1^, but it did not show deleterious activity against *Candida albicans* (Guzmán-Rodríguez et al., 2013). The tomato defensin TPP3 has a lipid binding specific for phosphatidylinositol (4,5)-bisphosphate (PIP2) with which it forms a dimeric configuration complex that is critical for membrane permeabilization and inhibits hyphal growth of *Fusarium graminearum* (Baxter et al., 2015).

### Lipid transfer proteins (LTPs)

The LTPs are small proteins with molecular masses lower than 10 kDa and rich in cysteine (Kader, 1996). They have about four to five α-helices, forming four disulfide bonds, which makes the LTP structure more stable, and thus more resistant to heat denaturation (Lindorff-Larsen et al., 2001; Berecz et al., 2010; Edstam and Edqvist, 2014). Hydrophobic residues of LTPs act by penetrating the membrane of the molecule, resulting in binding these hydrophobic molecules such as lipids (Finkina et al., 2016). LTPs can be expressed in fruits (Douliez et al., 2000; Carvalho and Gomes, 2007), and they may have an importance in plant survival, as well as in plant breeding (Salminen et al., 2016). Ca-LTP1, a peptide from chili pepper (*C. annuum* L.) seeds with molecular mass of 9461 Da, exhibited strong activity against the fungus *C. lindemunthianum, F. oxysporum, C. tropicalis*, and *C. albicans* and of the yeasts *S. cerevisiae, Schizosaccharomyces pombe*, and *Pichia membranifaciens* (Diz et al., 2006, 2011; Cruz et al., 2010). The peptide was further described as causing the formation of pseudohyphae and membrane disruption in *C. tropicalis*, with 70% of inhibition rate (Diz et al., 2011), morphological changes in *P. membranifaciens* and *C. albicans* and inhibition of the glucose-stimulated acidification of the medium in *S. cerevisiae* (Diz et al., 2006; Cruz et al., 2010).

### 2S albumins

2S albumins act as molecular reserves that are important for plant growth and defense mechanisms essential to plant survival. These proteins accumulate in storage vacuoles inside germinative tissues, such as seeds and kernels, and in vegetative tissues, such as tubercles and leaves. In response to pathogen attack, for example, 2S albumins could be synthesized in the form of a single large precursor polypeptide of 18–21 kDa and may be post-translationally modified by proteolytic cleaving. There is then the loss of a linker peptide and short peptides from both the N- and C-terminal, leading to the generation of two subunits of 8–14 and of 3–10 kDa (Ericson et al., 1986). These peptides have low molecular masses, presenting around 4–9 kDa (Hsiao et al., 2006; Maria-Neto et al., 2011), rich in glutamine and cationic residues (Youle and Huang, 1981; de Sousa Cândido et al., 2011) and well-known for their antimicrobial and antifungal activities (Ribeiro et al., 2011).

Pf2, a 2S albumin identified in seeds of *Passiflora edulis* f. flavicarpa, showed activity against pathogenic fungi of 24% for *F. oxysporum*, 32% for *Colletotrichum musae*, and the yeast *S. cerevisiae* and 78% for *C. lindemuthianum* (Agizzio et al., 2003). Pf2 inhibited conidial germination and hyphal elongation as well as inducing various hyphal morphological alterations in these fungi. The inhibition of the glucose-stimulated acidification of the incubation of *F. oxysporum* cells in 20 and 60 μg.mL^−1^, showed 20 and 40% inhibition, respectively, and interacted with the fungus' plasmatic membrane. Pf2 had homology with *Pe*-AFP1, another 2S albumin identified in seeds of *P. edulis*. *Pe*-AFP1 can inhibit fungal growth in *Trichoderma harzianum, Aspergillus fumigatus* and *F. oxysporum*, at rates of 80, 60, and 70% respectively, while not having activity against *Rhizoctonia solani, Paracoccidioidomicose brasiliensis*, and *C. albicans* (Pelegrini et al., 2006). Another 2S albumin found in *Passiflora alata* presented activity against the filamentous fungus *C. gloeosporioides*, but did not interfere with *Salmonella typhimurium* and *S. aureus* bacterial growth (Ribeiro et al., 2011). The bioassays carried out in these studies have boosted the search and identification of AMPs present in species of *Passiflora* (Ribeiro et al., 2011). Fractions of peptides present in chili pepper seeds (*C. annuum* L.) were able inhibit the growth of yeasts *S. cerevisiae, C. albicans, Candida parapsilosis, C. tropicali*s, *P. membranifaciens, K. marxiannus*, and *Candida guilliermondii* (Ribeiro et al., 2007). This fraction was also able to inhibit glucose-stimulated acidification of the medium by yeast cells of *S. cerevisiae* and to cause cell wall disorganization, bud formation, and formation of pseudohyphae.

### Glycine-rich proteins (GRPs)

GRPs are classified as storage proteins that are present in xylem, hypocotyls, stems, and petioles (de Sousa Cândido et al., 2011). This class contains abundant sequences of glycines in its primary structure and is hydrophobic due to its association with phenylalanine and tyrosine residues (Mousavi and Hotta, 2005).

The first report of a peptide of this class with activity against human pathogenic bacteria was done using a homodimer with molecular mass of 6029.34 Da isolated from *Psidium guajava* seeds. This peptide, named Pg-AMP1, had two α-helices, one at the N-terminus and another at the C-terminus, with a loop connecting them. Besides this, arginine residues provide a positive charge at the extreme of the helix, which can facilitate peptide/pathogen interactions (Pelegrini et al., 2008). According to Pelegrini et al. (2008), *Pg*-AMP1 can have a specific mode of action in prokaryotic bacterial cells, since it showed no inhibitory activity against the filamentous plant pathogenic fungi *T. harzianum, A. fumigatus, F. oxysporum*, and *R. solani* in the concentrations of 25, 50, 75, and 100 μg.mL^−1^, while revealing growth reduction against 90% of *Klebsiella* sp. and 30% of *Proteus* at a concentration of 40 μg.mL^−1^. Other peptides of the GRP class were isolated from *Coffea canephora* seed, named *Cc*-GRP (Zottich et al., 2013). These GRP peptides can cause changes in the membrane permeability of fungi *F. oxysporum* and *C. lindemuthianum* and prevent colony formation by yeasts. Besides this, the presence of the peptide in the cell wall, cell surface and nucleus of *F. oxysporum* suggests its action on different targets.

### Snakins

Snakins are a class of small peptides, ancient and ubiquitous, composed of 12 cysteines forming six disulfide bonds, with a highly conserved C-terminal, which is essential for their biological activity (Nahirnak et al., 2012; Mansour et al., 2014). Recently, one peptide was obtained from *Zizyphus jujuba* that is a fruit commonly used in popular medicine in Europe and Southeast Asia, having sedative, antitumor, analgesic and antipyretic properties, as well as being used as food (Pawlowska et al., 2009). Snakin-Z AMP, isolated from *Z. jujuba*, is 31 amino acid residues in length and has better activity againt fungi than bacteria (Terras et al., 1992; Thevissen et al., 1999; Selitrennikoff, 2001; Tran et al., 2002; Manners, 2009). Among bacteria, its action is stronger against Gram-negative than Gram-positive bacteria (Daneshmand et al., 2013).

SN2, a peptide found in tomato (*Solanum lycopersicum*), can perforate the biomembrane, with IC_50_ (drug concentration showing 50% inhibition) values of 2.17 ± 0.04 μM for hyphae and 8.02 ± 1.1 μM for microconidia cells, besideshaving strong microbicidal activity against *E. coli, Agrobacterium tumefaciens, Micrococcus luteus, Staphylococcus cohnii, P. pastoris, Fusarium solani*, with IC_50_ values between 0.9 ± 3 and 1.58 ± 0.24. This peptide could also be involved in an agglomeration effect in all tested microorganisms, which suggests that it could prevent the spread of pathogens out of the plant wound areas (Herbel et al., 2015).

### Napins

**Figure 1:**
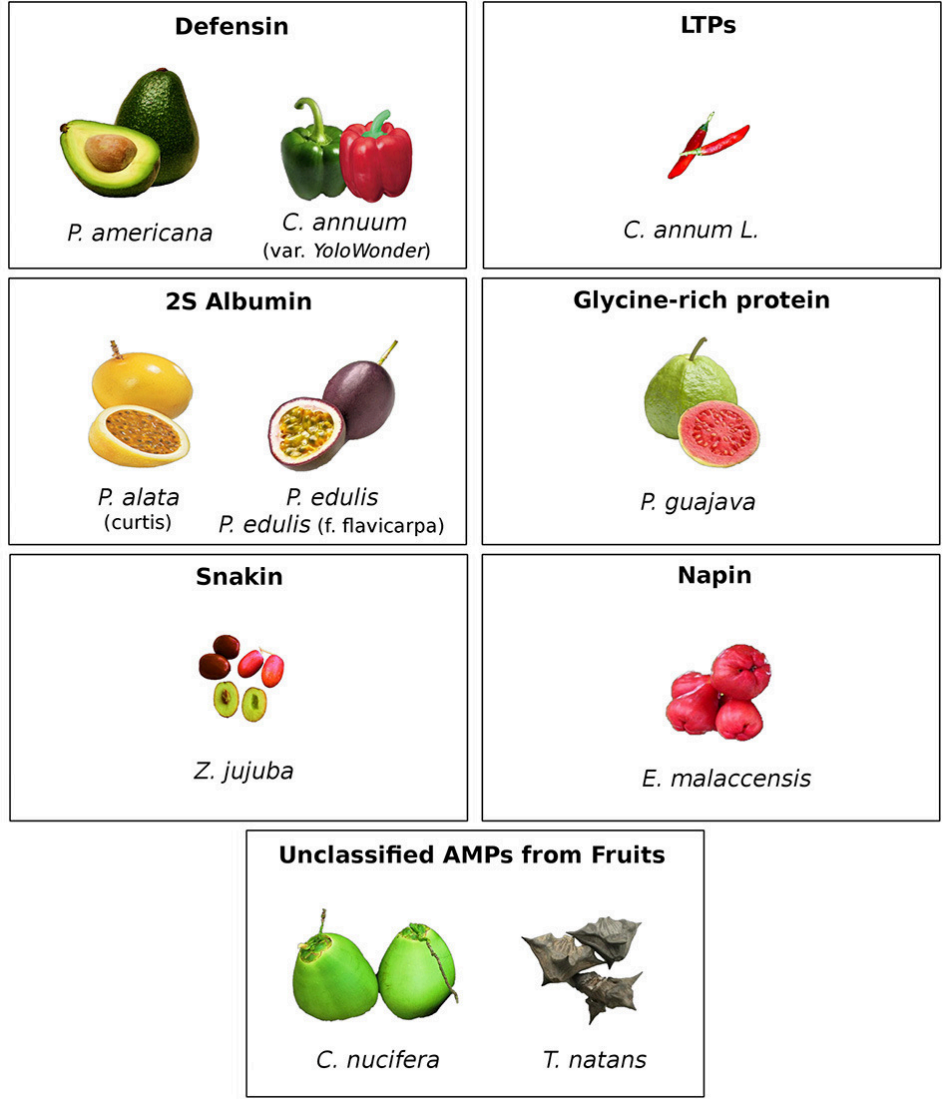
**Representative images of fruits containing antimicrobial peptides and indicative classes, which are Defensins (***P. americana, C. annuum*** var. Yolo Wonder); LTPs (***C. annum*** L.); 2S Albumin (***P. alata, P. edulis f. flavicarpa***); Glycine-rich protein (***P. guajava***); Snakin (***Z. jujuba***); Napin (***E. malaccensis***); and Unclassified AMPs from Fruit (***C. nucifera L., T. natans***)**.

Napins can present a low molecular mass, high water solubility and the presence of cysteine (Shewry et al., 1995). Normally, napins or napin-like proteins are synthesized as a single precursor (pre-pro napin) of about 12–15 kDa, which is processed into two polypeptide chains bound with disulfide bonds (Byczynska and Barciszewsk, 1999). The small polypeptide chain present in different napins and napin-like proteins ranges from ~3–4 kDa, while the large polypeptide chain is about 7–8 kDa (Shewry et al., 1995). Napins can be storage proteins synthesized during maturation, serving, in addition to their antibacterial, antifungal and trypsin inhibitor activities (Terras et al., 1992; Ng and Ngai, 2004; Ngai and Ng, 2004; Vashishta et al., 2006; Yang et al., 2007), as a source of nitrogen for germinating seedlings (Müntz, 1998; Ngai and Ng, 2003). (Da Silva Dantas et al., 2014) described the presence of a napin-type with molecular mass 1231.1 Da isolated in *Eugenia malaccensis*, named *Em2*, which showed inhibitory activity of 98 ± 0.9% against *S. aureus* and 40 ± 2.1% against *Salmonella enterica* of the Enteritidis strain (Figure 1).

### Unclassified AMPs from fruit

In this section, all AMPs will be described that are not covered in any class mentioned above. Wang and Ng (2005) isolated from coconut water a peptide with 10 kDa of molecular mass and antifungal activity against *F. oxysporum, Mycosphaerella arachidicola* and *Physalospora piricola*. In this study, the IC_50_ of *M. arachidicola* was 1.2 μM, inhibiting mycelial growth. Moreover, Mandal et al. (2009) described other peptides from green coconut (*Cocos nucifera* L.), but in this case with low molecular mass (858, 1249, and 950 Da). *Cn*-AMP 1, 2, and 3 were effective against Gram-positive and Gram-negative bacteria, but *Cn*-AMP1 had a better activity than the other two.

*Tn*AFP1, a peptide with molecular mass of 1230 Da and isolated from fruits of *Trapa natans*, showed the inhibition of *C. tropicalis* growth *in vitro* and inhibited the biofilm formation in a concentration dependent manner (Mandal et al., 2011).

The protein isolated from pokeweed (*Phytolacca americana*) is characterized by a pI higher than 10 and is homologous to the AMP from *Mirabilis jalapa*. The cDNA encoding the *P. americana* AMP (*Pa*-AMP-1) and the deduced amino acid sequence suggest that the protein is synthesized as a preprotein and secreted outside the cells, besides being present only in seeds (Liu et al., 2000). The positive patch and the hydrophobic surface of *Pa*-AMP can be essential to interact with the membrane of fungi *F. solani and Neurospora crassa* (Peng et al., 2005).

Other analyses using seeds extracted from *Litchi chinensis* and *Nephelium lappaceum* demonstrated different sizes of inhibition halos (mm) by disc diffusion method. The activity of *L. chinensis* against *Streptococcus pyogenes* (15 ± 0.55) followed by *Bacillus subtilis* (11 ± 0.00) and *S. aureus* (10 ± 0.25), and of *N. lappaceum* against *S. aureus* (13 ± 0.80) followed by *B. subtilis* (12 ± 0.40) and *S. pyogenes* (12 ± 0.10). However, *L. chinensis* extracts had activities against Gram-negative bacteria of 7.5 ± 0.30 and 9 ± 0.45 and *N. lappaceum* extract at 6.5 ± 0.66 and 10 ± 0.55 acted against *E. coli* and *Pseudomonas aeruginosa*, respectively. The MIC was determined in *S. pyogenes* strain to be the most sensitive, being 20 and 15 mg.mL^−1^ to *L. chinensis* and *N. lappaceum*.

## Biotechnological potential

The discovery of new groups of AMPs from fruit could provide a novel source of drug generation for the treatment of human infectious diseases (De Lucca, 2000; Hancock, 2000; Welling et al., 2000). In addition, the wide spectrum of antimicrobial activities in some molecules suggests they can have the potential for treating different types of cancer (Tanaka, 2001) as well as viral or parasitic infections (Wong et al., 2010).

According to a study performed by Hancock (2000), research with AMPs has been carried out for several years, demonstrating the important biotechnological development and its relevant therapeutic application, from topical administration to the systemic treatment of infections. Increasingly, AMPs are being consolidated as a new class of antibiotics, due to their broad mechanism of action, including against multi-resistant microorganisms.

AMPs act as a defense mechanism for living organisms, a fact that makes them a promising candidate for new antibiotic substances (Mansour et al., 2014). Still, it is expected that in a short time the defensins used in agro-products with antifungal activity will be used as an important instrument for the growth of agricultural production, a fact that should stimulate biotechnological research and the application of bioproducts (Lacerda et al., 2014).

AMPs with anti-infective activities have been developed in order to investigate their antimicrobial mechanisms, and it has been demonstrated that most AMPs act by interacting with the pathogen membrane (Harris et al., 2009; Lee and Lee, 2015; Dutta and Das, 2016; Lee et al., 2016). Chemical combination and modifications in targeted peptide residues are other options that can improve AMPs bioactivity, together with the use of biotechnology for the production of newly designed peptides in host plants or as biocontrol agents and for healthcare (López-García et al., 2000).

**Figure 2:**
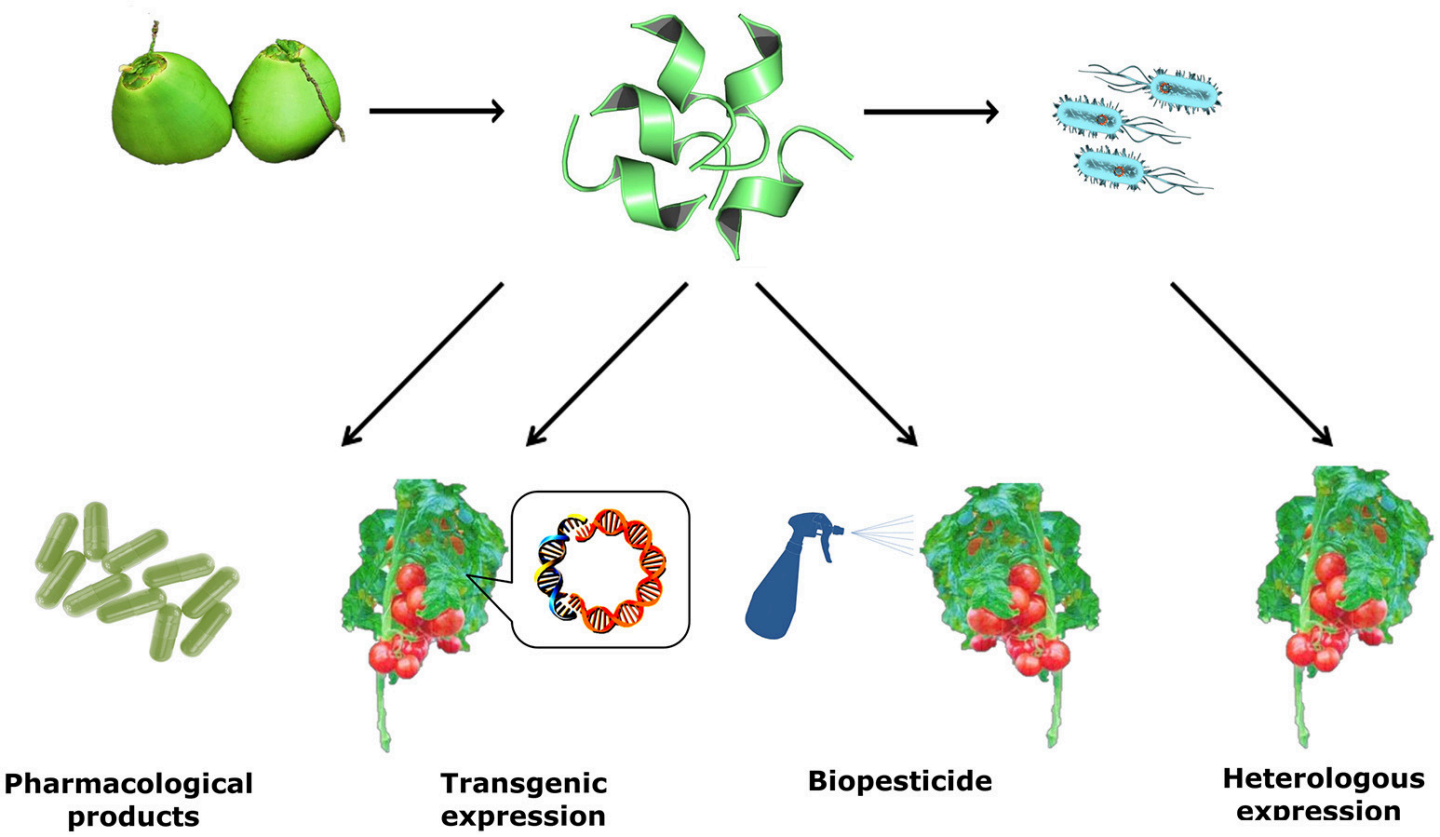
**Schematic representation of antimicrobial peptides isolated from fruits with promising pharmacological properties, representing a novel class of naturally occurring medicines**. The AMP is shown in this image (PDB: 2N0V) (Santana et al., 2015), a structural elucidation of a peptide isolated from green coconut water (*Cn*-AMP1) solved by solution NMR in the presence of SDS micelles, showing a helical content of 66.7%. Structure was visualized and edited on PyMOL version 1.4.1.

In the course of centuries, plants have been studied and serve as a major source of natural products and drugs. About half of the pharmaceutical products in use today are derived from natural products (Clark, 1996). Besides their therapeutic prospects, AMPs can be applied in the development of transgenic crops, decreasing the need for large quantities of pesticide in agriculture (Pelegrini and Franco, 2005). They have potential for conservation in the food industry (Yazdi et al., 2013) and in agribusiness (Meyer et al., 1996; Agizzio et al., 2003; Pelegrini et al., 2006, 2008; Ribeiro et al., 2011; Seo et al., 2014). Their medical use is likely to increase as anti-infective and immunomodulatory therapeutics (Matsuzaki, 1999; Mandal et al., 2009; Maróti et al., 2011; Silva et al., 2012, 2014; Jeong et al., 2015; Santana et al., 2015), in intracellular drug delivery, RNA, DNA, and nanoparticles in a non-destructive manner (Nasrollahi et al., 2012) (Figure 2).

Plant peptide screening has also introduced a new model to be applied to the development of crops resistant to pathogenic microorganisms, being of great interest in agribusiness (de Sousa Cândido et al., 2014). Moreover, the use of these molecules has effective fungicide action, besides producing lower impact on the environment compared to agrochemicals (Parachin and Franco, 2014). In this review, we have highlighted several studies regarding the antifungal properties of peptides isolated from fruits including J1, with action against *F. oxysporum, B. cinerea*, and *C. gloeosporioides* (Meyer et al., 1996; Seo et al., 2014); *Pa*-AFP1 against *C. gloesporioides* (Ribeiro et al., 2011); *Pe*-AFP1 against *T. harzianum, A. fumigatus*, and *F. oxysporum* (Pelegrini et al., 2006); *P*f2 against *F. oxysporum, C. musae, S. cerevisiae*, and *C. lindemuthianum* (Agizzio et al., 2003); and *Pg*-AMP against *T. harzianum, A. fumigatus, F. oxysporum*, and *R. solani* (Pelegrini et al., 2008).

Zainal et al. (2009) showed heterologous expression strategies of peptides in plants, and the isolation of these molecules from their natural source is presented as a strategy for AMP production. The expression of chili (*C. annuum*) defensins in tomatoes increased the tomato plant's resistance to *Fusarium* sp. and *Phytophthora infestans*, showing that transgenic lines could be more resistant to infection by these pathogens (Zainal et al., 2009). Moreover, some studies have demonstrated that transgenic expression of plant defensins leads to protection of vegetative tissues against pathogen attack, as is the case of *Fusarium*-resistant tomatoes expressing the *Ms*Def1 defensin gene (Thomma et al., 2002; Abdallah et al., 2010). For example, the rice chitinase gene (CHI), the alfalfa defensin gene (alfAFP), and their bivalent gene (CHI-AFP) (Chen et al., 2009) and the tobacco β-1,3-glucanase gene (GLU), alfalfa defensin gene alfAFP, and their bivalent gene GLU-AFP (Chen et al., 2006) were introduced into tomato line Micro-Tom via Agrobacterium-mediated gene transfer method. Besides these studies, overexpressing the defensin genes *Ms*Def1 *Medicago sativa* in crops of tomatoes proved that transgenic tomato lines exhibited higher resistance and dosage-effect to *B. cinerea, Ralstonia solanacearum*, and *F. oxysporum* f. *Lycopersici* sp. than that of non-transgenic plants (Abdallah et al., 2010). The floral defensin genes such as PhDef1, PhDef2, Sm-AMP-D1, and *DmAMP*1 were overexpressing in transgenic banana (Ghag et al., 2012, 2014) and papaya (Zhu et al., 2007) plants. These genes can improve resistance to phytopathogens such as *F. oxysporum* f. sp. cubense and *Phytophthora palmivora* when compared to untransformed control plants. Transformation with the *defensin* gene caused the reduced thickness of the hyphae cell wall of fungi in bioassays *in vitro* and *in situ* (Zhu et al., 2007). The presence of small defensin-like sequence genes (DEFL) in the grapevine genome can inhibit conidial germination of *B. cinerea* in this fruit (Giacomelli et al., 2012).

In addition to the promising application of these molecules in the development of innovative approaches in agriculture, we can see their future application in medicine, contributing to the improvement of biotechnological processes (Montesinos, 2007; Holaskova et al., 2015). According to Dutta and Das (2016), it is argued that resistance to AMPs is unlikely to resemble that found regarding conventional drugs. Hence, AMPs are a promising alternative in medicine. In order to elaborate and develop AMPs, making drugs with anti-infectious potential, there has been ample observation about their vast range of mechanisms of antimicrobial action, and of the way in which the interaction with the target membrane occurs in all mechanisms (Harris et al., 2009; Lee and Lee, 2015; Dutta and Das, 2016; Lee et al., 2016). However, there is still a need for more detailed studies, including *in vivo* studies using isolated plant molecules (Uhliga et al., 2014), due to certain pharmacological limitations and studies related to sensitivity, generating conflicting clinical analyses (Falagas and Kasiakou, 2005; Zavascki et al., 2007; Landman et al., 2008). However, the preparation of peptides with antibacterial activity is extremely expensive, which certainly makes clinical applicability difficult (Marr et al., 2006).

Plant AMPs have also been described as potential molecules in healthcare, being considered promising as therapeutic agents, and can be used as analogous peptides, thus boosting their therapeutic activity (Asthana et al., 2004; Sun et al., 2005). It has been proposed that plant AMPs, based on their broad-spectrum activity and efficiency, may offer a good alternative for the treatment of infections in relation to conventional antibiotics (da Silva and Machado, 2012). Some studies have shown the presence of different AMPs in fruits of *Capsicum* (Liu et al., 2006; Taveira et al., 2014) and avocado fruit (*Pa*Def), presenting antimicrobial activities that could be used in the treatment of infectious diseases caused by *E. coli* and *S. aureus* strains (Guzmán-Rodríguez et al., 2013). Moreover, the peptide *Ca*Thi associated with fluconazole showed inhibitory activities toward *C. albicans, C. tropicalis, C. parapsilosis, C. pelliculosa, C. buinensis*, and *C. mogii* (Taveira et al., 2016). Furthermore, it has been shown that plant AMPs cause an inhibitory effect on biofilm formation (Mandal et al., 2011), revealing that fruits could be excellent sources of bioactive molecules with promising health benefits (Skinner and Hunter, 2013).

Interestingly, an increasing number of works have labeled plant AMPs as promiscuous peptides, due to their different actions associated with the same structure (Franco, 2011). Among these are the peptide *Cn*-AMP1, considered multifunctional and promiscuous, presenting antibacterial and antifungal activity, also being capable to reduce the viability of tumor cells (Mandal et al., 2009; Silva et al., 2012; Santana et al., 2015). Another example is the peptides isolated from *Z. jujuba* fruits, with both antibacterial and antifungal properties against *S. aureus* and *Phomopsis azadirachtae*, respectively (Daneshmand et al., 2013). Extracts from fruits have also shown other activities, among these being antioxidant activity in the extract purified from *Z. jujuba*, which showed that some fractions can prevent oxidative reactions and are underutilized for preserving food and medicinal purposes (Yazdi et al., 2013). In addition, *Litsea japônica* fruits have been studied, showing that their extract can perform several activities, such as anti-osteoarthritis (Jeong et al., 2015) and activation of tumor necrosis factor-α (TNF-α) (Won et al., 2016).

Indeed, the study and description of new AMPs stemming from different parts of plants has proved to be a source of biotech products, investment in genetic engineering for the expression of these molecules, transgenic products already resistant to pathogens or production of insecticides in agriculture.

## Conclusions and outlook

In summary, the identification and isolation of various classes of peptides examined in this review details the importance of the antimicrobial activities found in these peptides from the seeds and pulp of fruits. They present promising applicability in the search for new medicines for human health, for ways to curb the spread of pests and for increasing production of fruits for agribusiness.

## Author contributions

BM from Universidade Católica Dom Bosco, Campo Grande–MS, Brazil, was responsible to write the topics Abstract and Fruit Antimicrobial Peptides Tables, furthermore contributes data to be inserted into the table. LM from Universidade Católica Dom Bosco, Campo Grande–MS, Brazil, contributed in Title, Abstract, Fruit Antimicrobial Peptides, Conclusion/outlooks and dates to insert into the table. KO from Universidade Católica Dom Bosco, Campo Grande–MS, Brazil was responsible to write the topic Biotechnological Potential and Figures. MN from Universidade Católica Dom Bosco, Campo Grande–MS, Brazil was responsible to write topic Fruit Antimicrobial Peptides and References formation. CC from Universidade Católica Dom Bosco, Campo Grande–MS, Brazil was responsible for the corrections of the manuscript. OF from Universidade Católica Dom Bosco, Campo Grande–MS, Brazil was responsible for the corrections of the manuscript.

### Conflict of interest statement

The authors declare that the research was conducted in the absence of any commercial or financial relationships that could be construed as a potential conflict of interest.
